# Blood Pressure Treatment Adherence and Control after Participation in the ReHOT

**DOI:** 10.5935/abc.20160165

**Published:** 2016-11

**Authors:** Nathália Silva de Jesus, Armando da Rocha Nogueira, Cacilda Oliveira Pachu, Ronir Raggio Luiz, Glaucia Maria Moraes de Oliveira

**Affiliations:** 1 Programa de Pós-Graduação em Cardiologia, Universidade Federal do Rio de Janeiro, RJ - Brazil; 2 Hospital Universitário Clementino Fraga Filho, Universidade Federal do Rio de Janeiro, RJ - Brazil; 3 Instituto de Estudos em Saúde Coletiva, RJ - Brazil

**Keywords:** Hypertension, Arterial Pressure, Medication Adherence, Clinical Trial, Antihypertensive Agents, Survey and Questionnaires

## Abstract

**Background:**

Lack of adherence to pharmacological treatment is one of the main causes of low control rates in hypertension.

**Objective:**

To verify treatment adherence and associated factors, as well as blood pressure (BP) control in participants of the Resistant Hypertension Optimal Treatment (ReHOT) clinical trial.

**Method:**

Cross-sectional study including all 109 patients who had completed the ReHOT for at least 6 months. We excluded those participants who failed to respond to the new recruitment after three phone contact attempts. We evaluated the BP control by ambulatory BP monitoring (ABPM; controlled levels: 24-hour systolic and diastolic BP < 130 x 80 mmHg) and analyzed the patients' treatment adherence using the Morisky Medication Adherence Scale (MMAS) questionnaire validated by Bloch, Melo, and Nogueira (2008). The statistical analysis was performed with the software IBM SPSS statistics 21.0. We tested the normality of the data distribution with kurtosis and skewness. The variables tested in the study are presented with descriptive statistics. Comparisons between treatment adherence and other variables were performed with Student's t test for independent variables and Pearson's chi-square or Fisher's exact test. To conduct analyses among patients considering adherence to treatment and BP control, we created four groups: G0, G1, G2, and G3. We considered a 5% significance level in all tests.

**Results:**

During the ReHOT, 80% of the patients had good BP control and treatment adherence. Of 96 patients reevaluated in the present study, only 52.1% had controlled hypertension when assessed by ABPM, while 31.3% were considered adherent by the MMAS. Regarding other ABPM measures, we observed an absence of a nocturnal dip in 64.6% of the patients and a white-coat effect and false BP control in 23% and 12.5%, respectively. Patients' education level showed a trend towards being a determinant factor associated with lack of adherence (p = 0.05). Resistant hypertension and number of medications were significantly associated with BP control assessed by ABPM (p = 0.009 and p = 0.001, respectively). Resistant hypertension was also significantly associated with group G0 (patients with no control or adherence, p = 0.012).

**Conclusion:**

There was a decrease in BP control and adherence measured by the MMAS after participation of at least 6 months in the ReHOT clinical trial.

## Introduction

Cardiovascular diseases are one of the leading causes of mortality worldwide and include hypertension as one of their most prevalent risk factors.^1^ According to the Global Burden of Disease, which evaluated the disease burden in 188 countries, hypertension was the second most important identifiable risk factor between 1990-2013, accounting for 10.4 million deaths.^2^ Hypertension has a high prevalence worldwide; it affects approximately 30 to 45% of the general population and increases sharply with age.^3^ In Brazil, the frequency of adults who reported a diagnosis of hypertension during a phone interview was 15.2% in Palmas and 30.7% in Rio de Janeiro.^4^

A study conducted in Ilha do Governador (Rio de Janeiro) between 1999 and 2009 to assess the cardiovascular mortality in hypertensive patients found a risk of cardiovascular death three times greater in hypertensive individuals compared with normotensive ones.^5^ Despite evidence regarding the effectiveness of antihypertensive treatment in reducing cardiovascular mortality and morbidity, the percentages of blood pressure (BP) control are very low. According to the VI Brazilian Guidelines on Hypertension (VI *Diretriz Brasileira de Hipertensão*, VIDBHA), these rates range from 20% to 40%.^1^ The BP control in hypertensive patients is directly related to adherence to the prescribed therapy, which in turn is one of the main factors responsible for uncontrolled hypertension.^6^

In Brazil, a review has shown rates of non-adherence of 49% in Rio de Janeiro and 25% in São Luiz between 2000 and 2009.^7^ Different factors interfering with this process include socioeconomic status, sex, age, education level, complexity of the therapeutic regimen, relationship with the health care team, and absence of symptoms.^8^ Lack of treatment adherence must not be confused with resistant hypertension, which is defined as the occurrence of BP levels above the target range (≥ 140 x 90 mmHg) despite the use of three antihypertensive drugs of different classes (including a diuretic at optimal doses).^9^ Patients with resistant hypertension must have good treatment adherence, since lack of adherence - namely pseudoresistance - may lead to addition of unnecessary medications to their treatment.

The Resistant Hypertension Optimal Treatment (ReHOT), a multicenter clinical trial in hypertensive patients conducted in 2010,^10^ aimed at identifying patients with resistant hypertension and standardize their therapeutic regimens. In this study, the rates of BP control and treatment adherence were approximately 80% (unpublished data).

Existing methods to evaluate treatment adherence may be classified as direct (including analytical measurements of the drugs metabolites or chemical markers that remain in the body for a longer time and identify whether the medication was administered or taken in the appropriate dosage and frequency) and indirect (including pill count, patient's report, physician's opinion, attendance to medical appointments, and use of validated questionnaires).^7,11^ Among the latter, the most commonly used are validated questionnaires, such as the Morisky and Green test, which comprises four questions (MGT4) to identify the attitude and behavior of the patient regarding medication use. One point is assigned to each negative response by the patient, and those who score four points are characterized as having adherence. Scores equal to or lower than three points characterize the patient as having no adherence.^12^ The MGT4 is considered a reference test for being a simple, validated instrument with easy application in clinical practice. It is the most often used test in studies evaluating treatment adherence.^7,13^

Assessment of treatment adherence is fundamental in the development of public and individual health care strategies. Based on that, the aim of this study was to evaluate the BP control, adherence to treatment, and factors related to non-adherence in patients who participated for at least 6 months in the clinical trial ReHOT.

## Methods

This was a cross-sectional study conducted between May 2014 and June 2015 in a cohort of hypertensive patients who had participated in the ReHOT clinical trial. The patients were recruited from the José Paranhos Fontenelle medical care unit, Manguinhos emergency care unit, and University Hospital Clementino Fraga Filho (HUCFF). The study included all patients who completed the ReHOT and excluded those who failed to respond to the new recruitment after three phone contact attempts.

The ReHOT was a multicenter, prospective, randomized study conducted between May 2011 and June 2013 in 26 centers in Brazil with the aim of identifying patients with resistant hypertension and standardizing their therapeutic regimens. The inclusion criteria were age between 18 and 75 years, systolic BP (SBP) ≥ 160 mmHg and ≤ 220 mmHg and/or diastolic BP (DBP) ≥ 100 mmHg, and regular enrollment in the participating center. The exclusion criteria were SBP > 220 mmHg, cardiovascular events (stroke and acute myocardial infarction) or cardiovascular procedures within the previous 6 months, stages IV and V renal insufficiency, NYHA functional class III and IV heart failure, history of malignant disease with a life expectancy < 2 years, alcoholism, psychiatric illness, lack of contraception (if women of childbearing age), pregnancy, severe arrhythmia, valvular heart disease, second- or third-degree atrioventricular (AVB) blockade without pacemaker, hyperkalemia (> 5.0 mEq/L), severe liver disease, renovascular disease and hyperaldosteronism, history of hypersensitivity to one of the medications included in the study protocol, grade III or IV fundoscopic changes, and requirement of use of a beta-blocker due to heart failure or coronary insufficiency. The research protocol of the study series was evaluated and approved by the research ethics committee of the coordinating center (INCOR), HUCFF, and by the Municipal Health Secretariat. The research ethics committee at HUCFF also approved a protocol amendment submitted for the study extension. All participants signed a free and informed consent form (protocol no. 189/09).

The ReHOT included 1927 individuals distributed across 26 participating centers. At HUCFF, a total of 123 individuals with stages II and III hypertension were recruited to estimate the prevalence of resistant hypertension in the first phase of the trial (visits 0 to 3). These patients were treated for 12 weeks according to recommendations by the Brazilian Guidelines on Hypertension^1^ (chlorthalidone 25 mg once a day, enalapril 20 mg twice a day, and amlodipine 5 mg once a day with potential increase to twice a day). Patients with a history of adverse reactions to enalapril or who presented adverse reactions to this medication during the trial were treated with losartan 50 mg twice a day. All patients were recommended to decrease sodium intake and practice physical activities, and were evaluated by a multidisciplinary team comprising physicians, nurses, and pharmacists. Treatment adherence was assessed by pill count during study appointments. During the study (visits 1 and 3), the patients underwent BP measurement, ambulatory BP monitoring (ABPM) and electrocardiogram (ECG), and were evaluated with routine laboratory tests (lipid profile, electrolytes, renal function, blood glucose, and urine sediment analysis). At visit 1, we conducted a special phenotypic characterization to identify those phenotypes with a predominance of increased renin-angiotensin system (RAS) activity or sympathetic activity, including measurement of urinary and plasma aldosterone, and 24-hour Na+ and K+ excretion. We also collected blood to organize a (serum) gene library and a biolibrary. In phase 2 (visits 4 to 5), the main objective was to evaluate a fourth drug to introduce in the therapeutic regimen to achieve control of the resistant hypertension. Those patients classified as having actual resistant hypertension were randomized to treatment with clonidine 0.100 mg twice a day (which could be titrated to 0.200 mg and 0.300 mg twice a day) and spironolactone 12.5 mg (which could be titrated to 25 mg and 50 mg). After 24 weeks, at the end of the study in visit 6, all routine laboratory tests, ECG, and ABPM were repeated for further evaluation.^10^

Of the 123 participants in the ReHOT clinical trial at HUCFF, 109 completed the study, 14 were excluded during the trial (three for not adjusting to the regimen due to white-coat effect [WCE], three due to loss to follow-up, two by the patients' own decision, two by the physicians' decisions, two due to adverse events not related to the medications, and two due to adverse events related to the medications). All 109 patients who completed the study were recruited by phone for the new evaluation and 13 were excluded after three unsuccessful contact attempts. The study included two visits (1 and 2). At visit 1, the patients filled out forms during medical and pharmaceutical appointments for collection of data identifying their sociodemographic characteristics (education level, race, sex, age, marital status, and occupation), comorbidities, alcoholism and smoking status, anthropometric measurements (weight and height), use of medications, and conventional BP measurement obtained according to recommendations by the VI DBHA^1^ (controlled BP: SBP and DBP < 140 x 90 mmHg) and placement of the ABPM device to determine the BP control (controlled BP: 24-hour SBP and DBP < 130 x 80 mmHg). ABPM was performed using with the oscillometric device Spacelabs Healthcare, model 90207 (ABP Report Management System, version 3.0.0.9). We considered as valid those ABPM tests obtaining 80% of the readings, and when this value was not reached, a new ABPM was obtained. BP measurements were obtained every 15 minutes during the day and every 20 minutes during the night, and the parameters used for the analysis were the DBP and SBP of the summary of the overall averages (values corresponding to the 24-hour period). A nocturnal dip (ND) was defined as a decrease in BP ≥ 10% from awake to sleep.^14^ A WCE was considered present when an individual had a BP outside the control target when measured at the office, but a normal ABPM. However, if the BP was controlled in the office but elevated during the ABPM, the patient was classified as having a false control.^13^

At visit 2, held on the day following visit 1, the ABPM device was removed and the patients then filled out the Morisky Medication Adherence Scale (MMAS) questionnaire, validated by Bloch et al.,^15^ to assess treatment adherence. A patient was considered treatment adherent when answering with a negative response to all questions.

The statistical analysis was performed with the software IBM SPSS statistics 21.0. The variables tested in the study are presented with descriptive statistics. The normality of the data distribution was tested with kurtosis and skewness; the distribution of the data was considered normal. Comparisons between treatment adherence and other variables were performed with Student's *t* test for independent variables, Pearson's chi-square test, or Fisher's exact test. We considered a 5% significance level in all statistical tests.

To perform analyses among patients considering adherence and BP control, we created four groups: group 0 (G0, without control and without adherence, comprising 32 patients), group 1 (G1, without adherence and with control, comprising 34 patients), group 2 (G2, with adherence and without control, with 14 patients), and group 3 (G3, with adherence and with control, with 16 patients).

## Results

Most of the 96 patients included in the study were non-whites. Their mean age was 53.9 years (26-76 years), 56.2% (n = 54) were women, 55.2% (n = 53) had been educated for up to 9 years, 57.3% (n = 55) had a partner, 17.7% (n = 17) were smokers, 36.5% (n = 35) were alcoholics, 27.1% (n = 26) were diabetics, and 30.2% (n = 29) were dyslipidemic. All patients had a prescription for at least two and up to four antihypertensive medications. In the overall cohort, 45.8% (n = 44) of the patients reported using two medications, 41.7% (n = 40) three medications, and 11.5% (n = 11) four medications. Only one patient reported not using any medication by his own decision although having a prescription for four medications. In total, 12.5% (n = 12) of the patients were classified as having resistant hypertension during the ReHOT trial (Table 1). Regarding the factors associated with treatment adherence, education level showed a trend towards being a determining factor (p = 0.05). The number of medications and the occurrence of resistant hypertension showed significant associations with BP control measured by ABPM (p = 0.009 and p = 0.001, respectively).

**Table 1:** General characteristics of the participants in the cross-sectional study

		With adherence % (n)	Without adherence % (n)	With control % (n)	Without control
Sex					
Female		28 (15)	72 (39)	53.7 (29)	46.3 (25)
Male		35.7 (15)	64.3 (27)	50 (21)	50 (21)
Age in years					
	Mean	52.92 ± 10.3	55.97± 9.29	54.6 ± 9.3	53.09 ± 10.8
Smoking					
Yes		17.6 (3)	82.3 (14)	64.7 (11)	35.5(6)
No		34.2 (27)	65.8 (52)	49.4 (39)	50.6 (40)
Alcoholism					
Yes		22.9 (8)	77.1 (27)	65.7 (23)	34.3 (12)
No		36 (22)	64 (39)	44.3 (27)	55.7(34)
Marital status					
With a partner		30.9 (17)	69.1 (38)	52.7 (29)	47.3 (26)
Without a partner		31.7 (13)	68.3 (28)	52.2 (21)	48.8 (20)
Diabetes					
Yes		30.8 (8)	69.2 (18)	46.2 (12)	53.8 (14)
No		31.5 (22)	68.5 (48)	54.3 (38)	45.7 (32)
Dyslipidemia					
Yes		34.5 (10)	65.5 (19)	48.3(14)	51.7 (15)
No		29.9 (20)	70.1 (47)	53.7 (36)	46.3 (31)
Education level^+^					
0 - 9		22.7 (12)	77.3 (41)	51.5 (31)	41.5 (22)
> 9		41.9 (18)	58.1 (25)	44.2 (19)	55.8 (24)
Self-reported ethnicity					
White		49.8 (11)	59.2 (16)	48.1 (13)	51.9 (14)
Non-White		27.5(19)	72.5 (50)	53.6 (37)	46.4 (32)
Number of medications^*^					
Mean ± SD		33.3(4)	66.7(8)	8.3 (1)^*^	91.7 (11)
Resistant hypertension^+^					
Yes		33.3(4)	66.7(8)	8.3 (1)^*^	91.7 (11)
No		31 (26)	69 (58)	58.3 (49)	41.7 (35)
Time after the Rehot					
Mean		19±8.2	19.1±8.6	18.3±8.3	19.9±8.5

Significância:

* p < 0,05 -

+ p = 0,05

Overall, 52.1% (n = 50) of the 96 patients evaluated in the present study were identified as having controlled hypertension (< 130 x 80 mmHg) when assessed by 24-hour ABPM. The corresponding rate of BP control at the end of the ReHOT trial was 79.2% (n = 76).

Taking into consideration other ABPM measures, we found that 64.6% (n = 62) of the patients had no ND, 23% (n = 22) had WCE, and 12.5% (n = 12) had a false BP control (Table 2).

**Table 2:** Association between blood pressure control measured in the office versus ambulatory blood pressure monitoring (ABPM) and adherence to treatment, as measured by the Morisky and Green questionnaire validated by Bloch et al^15^

CONTROL OFFICE BP			ADHERENCE AND MORISKY AND GREEN		
			No	Yes	Total
No	Control 24-h ABPM	No	23	11	34
		Yes	16	6	22
					
Total	46		39	17	56
					
					
Yes	Control 24-h ABPM	No	9	3	12
		Yes	18	10	28
					
Total	50		27	13	40
					
Total			66	30	96

ABPM: Ambulatory Blood Pressure Monitoring; BP: Blood Pressure.

Of the 96 patients who completed the study, only 31.3% (n = 30) had treatment adherence according to the MMAS. An analysis of the association between adherence and ABPM values showed that 16.7% (n = 16) had treatment adherence and BP control, while 14.6% (n = 14) had treatment adherence but no BP control. Regarding lack of treatment adherence, 35.4% (n = 34) of the patients failed to achieve BP control and 33.3% (n = 32) failed to show treatment adherence, despite presenting BP control when assessed with the ABPM (Table 3).

**Table 3:** Association between blood pressure control, assessed by ambulatory blood pressure monitoring (ABPM), and adherence to treatment, measured with the Morisky and Green questionnaire validated by Bloch et al^15^

	Blood pressure control assessed by ABPM*		
	Yes	No	Total
	%	%	
With adherence	16.7(16)	14.6(14)	31.3 (30)
Without adherence	35.4(34)	33.3(32)	68.7 (66)
Total	52.1 (50)	47.9 (46)	

* Blood pressure control assessed by the ABPM: 24-hour systolic and diastolic blood pressure < 130 x 80 mmHg.

When we evaluated the results of the questionnaire by subgroup of patients, we identified that among 12 patients regarded as having a false control, 75% (n = 9) had well-controlled office BP but lacked control when the BP was assessed by ABPM. In another subgroup comprising patients with resistant hypertension, we observed that 91.6% (n = 11) of the patients lacked control when their BP levels were assessed with ABPM and 66.7% (n = 8) were non-adherent (Table 2).

Table 4 shows the relationship between the factors considered influential on treatment adherence and the groups of patients according to adherence and BP control by ABPM. We observed that the female sex was the most prevalent in all groups (G0, G1, G2, and G3) and that the average age differed among the groups and was higher in G3 (56.19 ± 9.0 years). Most patients in each group were non-whites, were smokers or alcoholics, had a partner, and had no diabetes, dyslipidemia, or hypertension. The average number of prescribed medications was greater in G2 (2.92 ± 0.83). Group G0 had more patients with resistant hypertension (p = 0.012) and a longer mean follow-up after the ReHOT trial (20.4 ± 9.2 months). The education level differed between the two first groups (G0 and G1), in which most patients received education for up to 9 years, and the last two groups (G2 and G3), in which the patients received education for more than 9 years.

**Table 4:** General characteristics of the patients who participated in the cross-sectional study divided by groups

		G0Without adherence / Without control	G1Without adherence / With control	G2 With adherence / Without control	G3With adherence / With control	Total
		**% (n)**	**% (n)**	**% (n)**	**% (n)**	**% (n)**
**Sex**						
Female		33.3 (18)	38.9 (21)	13 (7)	14.8 (8)	56.3 (54)
Male		33.3 (14)	31 (13)	16.7 (7)	19 (8)	43.8(42)
**Age in years **						
	Mean	51.9± 11.1	53.8 ±9.5	55.7±9.9	56.19±9.0	53.8±10
**Smoking**						
Yes		35.3(6)	47.1 (8)	-	17.6 (3)	17.7 (17)
No		32.9 (26)	32.9 (26)	17.7 (14)	16.5 (13)	82.3 (79)
**Alcoholism**						
Yes		31.4 (11)	45.7 (16)	2.9 (1)	20 (7)	36.5 (35)
No		34.4 (21)	29.5 (18)	21.3 (13)	14.8 (9)	63.5 (61)
**Marital status**						
With a partner		32.7 (18)	36.4 (20)	14.5 (8)	16.4 (9)	57.3 (55)
Without a partner		34.1 (14)	34.1 (14)	14.6 (6)	17.1 (7)	42.7 (41)
**Diabetes**						
Yes		34.6(9)	34.6 (9)	19.2 (5)	11.5 (3)	27.1 (26)
No		32.9 (23)	35.7 (25)	12.9 (9)	18.6 (13)	72.9 (70)
**Dyslipidemia**						
Yes		34.5 (10)	31 (9)	17.2 (5)	17.2 (5)	30.2 (29)
No		32.8 (22)	37.3(25)	13.4 (9)	16.4 (11)	69.8 (67)
**Education level**						
0 - 9		32.1 (17)	45.3 (24)	9.4 (5)	13.2 (7)	55.2 (53)
> 9		34.9 (15)	23.2 (10)	20.9 (9)	20.9 (9)	44.8 (43)
**Self-reported ethnicity**						
White		33.3 (9)	25.9 (7)	18.5 (5)	22.2 (6)	28.1 (27)
Non-White		33.3 (23)	39.1 (27)	13 (9)	14.5 (10)	71.9 (69)
**Number of medications**						
	Mean	2.78±0.87	2.5±0.56	2.92±0.83	2.31±0.48	2.62±0.72
Resistant hypertension*						
Yes		58.3 (7)	8.3 (1)	33.3 (4)	-	12.5 (12)
No		29.8 (25)	39.3 (33)	11.9 (10)	19 (16)	87.5 (84)
**Time after rehot**						
	Mean	20.4±9.2	17.8± 8.0	18.9±7.0	19.1±9.27	19.0±8.4

Significance:

* p < 0.05

Even though the rate of adherence was lower than that of non-adherence, most patients (55.2%, n = 53) reported not having problems to remember taking their medications. Regarding treatment interruption, 85.4% (n = 82) and 85.7% (n = 84) reported not stopping their medications when feeling better or worse, respectively (Table 5).

**Table 5:** Association between answers to the Morisky and Green questionnaire validated by Bloch et al^15^ and total adherence according to the questionnaire

Questions	Yes (n)	No (n)	Adherence % (n)
1. Do you sometimes have trouble remembering to take your medication?	43	53	43.4 (23)
2. Do you sometimes neglect to take your medication?	49	47	63.8 (30)
3. When you are feeling better, do you sometimes stop taking your medication?	14	82	36.6 (30)
4. If you feel worse when taking the medication, do you sometimes stop taking it?	12	84	23.8 (30)

## Discussion

A few studies^16-18^ have correlated some variables with treatment non-adherence and consequently lack of hypertension control, including sex, age, education level, race, marital status, smoking, alcoholism, presence of comorbidities, length of follow-up, and number of medications in use. In this study, education level showed a trend towards being a determinant factor associated with lack of treatment adherence. Although the literature lists education level as being a possible factor in treatment adherence, only a few studies have found a direct association between both. Martins et al.,^19^ in a study evaluating treatment adherence in hypertensive patients using the MMAS, found no significant association between education level and adherence, although the population in their study comprised mostly individuals with low education level. The small number of individuals evaluated in our study (n = 96) probably influenced this result. This issue is clinically important since it impacts the patient's understanding of the recommendations. Individuals with low education level have greater challenge in understanding medical prescriptions, package inserts, and information communicated by their health care professional.^19^

Another factor described in the literature regarding treatment adherence is the number of prescribed medications. In our study, there was no significant association between the number of medications and treatment adherence, although we found a significant relationship between the number of medications and lack of BP control. The higher the number of medications used, the greater the risk of interactions and adverse reactions, resulting in decreased treatment adherence and, consequently, worse BP control. Therefore, treatment simplification is an important strategy to improve adherence and BP control.^13,20^

The present study found a significant association between resistant hypertension and BP control assessed by ABPM. Of the 12 patients with resistant hypertension in the cohort, 11 lacked BP control. Of these, eight were considered non-adherent by the MMAS, which brings up an important issue, since by not adhering to the treatment, these patients cannot be considered actually resistant. This imposes a decision to either increase the number of prescribed medications or introduce strategies to improve adherence.

When we evaluated the influence of factors related to adherence in different groups of patients, resistant hypertension also had a significant association with G0, which comprised patients without control or treatment adherence. A study conducted in 2008 by Bloch et al.^15^ used three assessment methods (including the MMAS) to analyze adherence and BP control in patients with resistant hypertension using conventional office measurement and ABPM. These authors observed a decrease in BP measured both by a conventional method and ABPM in patients considered adherent when the BP was measured by any of the methods.

A correct diagnosis of resistant hypertension must take into account the treatment, adherence to treatment, and BP control since patients without adherence and consequently, lack of BP control may be confused with those with truly resistant hypertension and undergo unnecessary tests and prescription modifications.^9^ The patients with resistant hypertension in our sample were identified after careful evaluation of BP control using ABPM during the ReHOT trial, when they were appropriately medicated. It is possible that when the trial ended, the patients returned to behave similarly to others in general, decreasing treatment adherence and hypertension control.

The comparison of studies with patients with resistant hypertension is a difficult task, given that only a few studies consider treatment adherence when evaluating the diagnosis and differentiation of pseudoresistance. Oliveira-Filho et al.,^21^ in a study to evaluate the adherence of hypertensive patients and identify patients with resistant hypertension, observed that lack of adherence was an important problem among the patients, and more than half of the study patients could have been diagnosed with pseudoresistant hypertension caused by lack of adherence.

Regarding BP control, we observed that only 52.1% (n = 50) of the patients had controlled hypertension when assessed by ABPM, showing that the control rate decreased soon after participation in the ReHOT trial, when the rate was 79.2% (n = 76). The low control rate found in our study is consistent with other recent studies, including one conducted in 2012 at a primary health care center in Rio Grande do Sul, in which 55.2% of the patients had controlled hypertension assessed by 24-hour ABPM,^20^ and another study conducted by Guimarães Filho et al.^22^ at a hypertension and diabetes referral center in Goiás, which found an even lower BP control rate (39.6%) when the BP was measured by a conventional method. These studies suggest that regardless of the method used for BP measurement, the main reason for low BP control rates is the relationship between patients and health care professionals, emphasizing a need for multidisciplinary teams to improve the service provided to hypertensive patients.

Mori et al.^23^ observed a higher rate of hypertension control in patients advised about medication use by a multidisciplinary health care team; awareness was considered a motivation for adherence to the recommendations, demonstrating that education improves clinical response. The interruption of the multidisciplinary care received during the ReHOT trial may have strongly influenced the decrease in BP control rate observed in these patients, since during the trial these patients received in addition to medications, information about their disease and healthy lifestyle habits.

The adherence rate of 31.3% (n = 30) found in the present study corroborates the findings of the study by Bastos-Barbosa et al.,^13^ in which the adherence rate of hypertensive patients evaluated by the MMAS was 36%. Of possible factors associated with poor adherence to antihypertensive treatment, access to medications is an important one. However, only the provision of medications does not guarantee that these medications will be used correctly; there is need for a therapeutic follow-up by qualified professionals, particularly for those patients who tend to be non-adherent.^24^

The MMAS is the most commonly used questionnaire to evaluate lack of adherence to pharmacological treatment, due to its feasibility, practical application, and low cost.^19^ Although we are unable to compare the adherence results obtained with the questionnaire with those obtained by pill count, as conducted in the ReHOT, we observed a decrease in the adherence rate when compared with that found during the clinical trial, which was 80%.

During the ReHOT, the therapeutic regimen was standardized, and treatment adherence was verified during study appointments. The patients were also instructed about medication use and possible adverse events by pharmacists who were part of the team. Even though the patients continued to receive most medications free of charge from the Popular Pharmacy Program, their return to the conventional health care system may have weakened the relationship established during the ReHOT trial, causing the adherence rates to decrease.

According to a systematic review published in 2014 by Matthes et al.,^25^ effective measures to improve adherence include the provision of information (positive and negative) to the patients regarding their disease and treatment, active integration of the patients, as well as consideration of factors that affect adherence in general and personal possibilities and needs.

When we considered other measures evaluated with the ABPM, we found a 23% (n = 22) frequency of WCE. According to the Department of Hypertension (DHA) of the Brazilian Society of Cardiology,^26^ WCE is one of the causes of pseudoresistance and ABPM is an important tool to establish its diagnosis. This fact emphasizes the importance of measuring BP by ABPM to identify these patients, thus preventing them from being wrongfully characterized.

As in other studies,^13,20^ we observed no direct association between adherence to antihypertensive treatment, as measured by the MMAS, and controlled BP, assessed by 24-hour ABPM. Although the questionnaire failed to show a good performance in general, it identified one-third of the patients as being adherent to the therapy. Furthermore, the results showed that in patients' subgroups, the questionnaire had the ability to correlate the occurrence of uncontrolled BP with a negative attitude towards the use of the antihypertensive medications. They also demonstrate that patients with hypertension require routine use of methods to measure adherence, such as the MMAS, and ABPM assessments. Without these measures in combination, patients like these could be considered as having well-controlled hypertension and continue with elevated BP levels and increased risk of cardiovascular events. Alternatively, patients with resistant hypertension would have their medications doses or amounts increased without need, resulting in unnecessary costs and adverse effects.

The results of this study suggest that the association of a method evaluating adherence with another assessing BP levels outside the office to evaluate the control of hypertension would help the health care system improve the identification of patients who require greater attention to achieve their control goals. Considering the possible challenges in implementing such methods in the health care system, these assessments could be initiated in small groups. Hypertensive patients with well-controlled office BP who are classified as non-adherent to treatment, for example, would be the group of first choice for evaluation with ABPM.

Although this study used two different methods to compare patients' adherence at two time points, the comparison of the BP control by ABPM at both moments was standardized and showed worse BP control in parallel with decreased adherence. Although the loss of 13 (11.9%) of the 109 patients who concluded the ReHOT trial was small, it may have influenced the lack of significance found in the analysis of some variables such as education level, which showed a borderline significance.

## Conclusion

The results of this study show a reduction in BP control rates measured by ABPM and adherence to hypertension treatment assessed with the MMAS with at least 6 months of participation in the ReHOT clinical trial. Regarding the factors associated with treatment adherence, education level showed a trend towards being a determining factor. Resistant hypertension and the number of prescribed medications had no significant association with the BP measured by ABPM. When we analyzed the same factors by patient group, we found that resistant hypertension also had a significant association with G0. The results also suggest that there is a need in the health care system of implementation of means to determine the patients' adherence to pharmacological treatment in combination with a method to measure the BP outside the office. The combination of these two assessments would allow a the diagnosis of hypertensive situation identifying whether or not it is controlled of hypertensive patients and development of strategies to improve BP control.
